# Low-Intensity Resistance Exercise Combined With Blood Flow Restriction is More Conducive to Regulate Blood Pressure and Autonomic Nervous System in Hypertension Patients—Compared With High-Intensity and Low-Intensity Resistance Exercise

**DOI:** 10.3389/fphys.2022.833809

**Published:** 2022-04-20

**Authors:** Yan Zhao, Yuchan Zheng, Xiaohuan Ma, Lili Qiang, Aicui Lin, Mo Zhou

**Affiliations:** ^1^ School of Sports and Health, Nanjing Sport Institute, Nanjing, China; ^2^ Rehabilitation Hospital of Huishan District of Wuxi, Wuxi, China; ^3^ Department of Science and Technology, Nanjing First Hospital, Nanjing Medical University, Nanjing, China; ^4^ Department of Rehabilitation, Nanjing First Hospital, Nanjing Medical University, Nanjing, China

**Keywords:** hypertension, autonomic nervous system, resistance exercise, blood flow restriction, blood pressure

## Abstract

**Background:** The effect of resistance exercise on the autonomic nervous system of patients with hypertension has not been identified.

**Objective:** To explore a suitable resistance training method for hypertension patients to regulate blood pressure (BP) and autonomic nervous system function.

**Method:** Forty-five hypertension patients aged between 55 and 70 years were randomly equally divided into three groups: the high-intensity resistance exercise (HE) group, the low-intensity resistance exercise combined with blood flow restriction (LE-BFR) group, and the low-intensity resistance exercise (LE) group. All patients performed quadriceps femoris resistance exercise. The exercise intensity of HE, LE-BFR and LE group was 65, 30 and 30% of one repetition maximum (1RM), respectively. The LE-BFR group used pressure cuffs to provide 130% of systolic pressure to the patient’s thighs during resistance exercise. The training program was 20 times/min/set with a 1-min break after each set, and was conducted five sets/day and 3 days/week, lasting for 12 weeks. The heart rate (HR), BP, root-mean-square of difference-value of adjacent RR intervals (RMSSD), low frequency (LF) and high frequency (HF) were evaluated before and after the first training and the last training.

**Result:** Significant differences in HR were observed in both recovery states after the first and last training (*p* < 0.01). After 12 weeks of training, the recovery speed of HR in the LE-BFR group increased significantly (*p* < 0.01). The systolic blood pressures in the HE and LE-BFR group were significantly reduced (*p* < 0.05 and *p* < 0.01), and the differences among groups were significant (*p* < 0.01). In the last recovery state, the RMSSD of the LE group was significantly lower than that in the first recovery state (*p* < 0.01). The LF/HF ratios of the HE and LE groups in the resting and recovery states were increased significantly (all *p* < 0.01). LF/HF ratios in the LE-BFR group in the resting and recovery state were decreased significantly (both *p* < 0.01).

**Conclusion:** Compared to HE and LE, LE-BFR could effectively decrease systolic pressure and regulate the autonomic nervous system function in hypertension patients.

## 1 Introduction

Hypertension is an important public health problem worldwide and is considered the main risk factor for cardiovascular diseases (Mozaffarian et al., 2015). The autonomic nervous system (ANS) plays an important role in regulating blood pressure (BP). Studies have found that imbalance and dysregulation of the ANS, manifested as enhanced sympathetic activity (Li et al., 2020; Esler, 2011), precede the occurrence of hypertension and develop concurrently with hypertension (Hering et al., 2016; Mancia and Grassi, 2014; Zubcevic et al., 2019). Therefore, it is important to modify the imbalance and dysregulation of the ANS for hypertension patients with sympathetic predominant states.

Exercise helps to lower BP, but patients with arterial hypertension were advised to avoid high levels of acute cardiovascular stress (Vale et al., 2018) because of the sympathetic predominance. Even though aerobic exercise was demonstrated to be able to inhibit sympathetic predominance (Esler, 2011), the effect of resistance exercise on decreasing sympathetic activity has not yet been identified in previous studies (Trevizani et al., 2018).

A transient research has found high-intensity resistance exercise (HE) can lead to a significant increase in systolic blood pressure (SBP) after the exercise, while low-intensity resistance exercise (LE) cannot, but LE can lead to a significant increase in the low frequency/high frequency (LF/HF) ratio (Vale et al., 2018). Hence, it remains unclear whether high-intensity and low-intensity exercise are unfavorable to patients with hypertension, and the exploration of resistance exercise suitable for hypertensive patients is required to reduce BP and avoid cardiovascular disease risks.

Low-intensity resistance exercise combined with blood flow restriction (LE-BFR) has been widely implemented to investigate its cardiovascular effect, but most of the research explored the cardiovascular effect on healthy adults. Early et al. found LE-BFR could lower SBP in healthy young adults (Early et al., 2020). However, studies on LE-BFR in hypertension patients are rare (Cerqueira et al., 2021).

This study aimed to analyze and compare the impacts of HE, LE, and LE-BFR on BP and ANS in hypertension patients, and to provide the most efficacious resistance exercise procedure that could reduce BP and avoid cardiovascular disease risks for hypertension patients.

## 2 Methods

### 2.1 Participants

Forty-five hypertension patients aged between 55 and 70 years, including 16 men and 29 women, were voluntarily included. Patients with the following features were excluded: 1) body mass index (BMI) > 28; 2) abnormal electrocardiogram in the resting state and after exercise; 3) with musculoskeletal disorders; 4) with physical exercises more than twice a week; 5) SBP ≥180 mmHg and/or diastolic blood pressure (DBP) ≥ 100 mmHg in resting state; 6) special diet control. Patients were included in this study only when the clinical evaluation showed that they had no restrictions on participating in physical exercise. A written informed consent was obtained from each patient after they understood the detailed description of all procedures. This study was approved by the Ethics Committee for Human Experiments of Rehabilitation hospital of Huishan in Wuxi, Jiangsu, China (ID: HK-LLWYH-202002).

### 2.2 Trial Design

#### 2.2.1 Groups

Patients were randomly equally divided into three groups: HE, LE-BFR, and LE groups.

#### 2.2.2 One Repetition Maximum

The one repetition maximum (1RM) of quadriceps femoris was evaluated by the Isokinetic Muscle Strength Evaluation Training System (SYSTEM4, BIODEX Co., Ltd., New Jersey, United States). Before the evaluation, each patient underwent a 15 minutes of lower limb muscle stretching as warm-up and then sat on the chair of the isokinetic dynamometer with one lower leg perpendicular to the ground. Before the evaluation, all patients participated in an adaptation session. When doing adaptation exercises, the patients performed ten contractions of quadriceps femoris to move the knee joint from 90° flexion to 0° extension without load. During the evaluation, the patients performed three contractions of quadriceps femoris to move the knee joint from 90° flexion to 0° extension. The instrument calculated the 1RM by the strength of contraction. The 1RM of both legs was evaluated, and the higher value was selected as the patient’s evaluation result. An assessment was conducted every 4 weeks to help the subjects adjust their exercise intensity.

#### 2.2.3 Measurement of Cardiac ANS Function

The autonomic modulation index of the cardiovascular system was obtained by FIRSTBEAT (Version 4.7.3.1, Firstbeat Technologies Ltd., Jyväskylä, Finland). Time domain variables examined included the mean heart rate (HR) and the root-mean-square of difference-value of adjacent RR intervals (RMSSD). Frequency domain indicators examined included low frequency (LF; 0.04–0.15 Hz) and high frequency (HF; 0.15–0.50 Hz) values. In addition, the LF/HF ratio was calculated to represent the sympathovagal balance (Task Force, 1996).

Participants were required to avoid drinking any liquids containing caffeine and/or alcohol within 2 h before the assessment. All evaluations were performed at least 2 h after a meal. The index of ANS in the resting states were obtained 0–15 min before the first and the last trainings. In order to get the indicators of ANS in the recovery state on the same time period, RMSSD, LF and HF were obtained 10–20 min after the first and the last trainings. HR in the recovery state was collected 10–30 min after the training, as it turned stable 10 min after the training.

#### 2.2.4 Blood Flow Restriction Intervention

Blood pressures were measured 0–15 min before the first and last trainings in the resting state. For patients in the LE-BFR group, inflatable pressure cuffs (KAATSUMASTER, Kaatsu Japan Co., Ltd., Tokyo, Japan) were used to wrap around the upper third of both thighs to restrict blood flow. When patients performed exercises, the pressure cuffs were inflated and the pressure was 130% of the subjects’ SBP (Zhao et al., 2021). The pressure cuffs were removed after each set of exercise.

### 2.3 Training Program

The resistance exercises in each group were conducted by contracting quadriceps femoris, leading to the extension of knee joints on the isokinetic dynamometer. The exercise intensity of patients in the HE group was 65% of 1RM, and that in the LE group and LE-BFR group was 30% of 1RM. During exercises, patients in the LE-BFR group underwent blood occlusions by inflating the pressure cuffs. The resistance exercise program was 20 times/min/set, with a 1-min break after each set and a number of five sets/day. The exercise frequency was 3 days/week, for a total of 12 weeks. Each repetition was completed within 3 s, with the rhythm controlled by a metronome. Both legs of the patients were trained and their daily physical activities were not intervened.

### 2.4 Statistical Analyses

The measurement data were expressed as mean ± standard deviation (SD). Differences within each group were analyzed using the paired *t* test. Differences among three groups were analyzed by one-way ANOVA, and the LSD method was used for multiple comparisons. The *p*-value reported was two-sided, and the differences with a *p*-value <0.05 was considered statistically significant, while *p*-value <0.01 was considered highly statistically significant. All statistical analyses were performed using SPSS software (version 13.0, SPSS, Chicago, IL, United States).

## 3 Results

### 3.1 Basic Characteristics

Before the training started, there were no significant differences among groups in terms of age, height, body mass, and other characteristics (Table 1).

**TABLE 1:** Characteristics of participants included in the study.

Variables	LE group	LE-BFR group	HE group
Age (years)	61.0 ± 4.3	63.0 ± 5.2	61.7 ± 3.6
Height (m)	158.7 ± 5.4	160.7 ± 3.5	158.0 ± 8.4
Body mass (kg)	65.0 ± 10.1	60.0 ± 6.2	63.9 ± 8.6
Body mass index (kg/m^2^)	25.7 ± 2.9	23.2 ± 2.0	25.5 ± 2.7
1RM (kg)	38.2 ± 2.5	36.4 ± 4.7	39.8 ± 3.9
Heart rate (b/min)	72.0 ± 6.7	73.0 ± 4.8	69.9 ± 6.6
SBP (mmHg)	145.9 ± 9.3	144.7 ± 7.9	148.1 ± 11.4
DBP (mmHg)	77.2 ± 5.5	74.5 ± 5.4	75.9 ± 9.8
Fasting blood glucose (mmol/L)	6.5 ± 1.2	6.0 ± 1.1	6.0 ± 0.8
Total cholesterol (mmol/L)	4.7 ± 1.0	5.0 ± 1.3	4.9 ± 0.7
Triglyceride (mmol/L)	1.7 ± 1.0	1.6 ± 0.8	1.8 ± 0.9
Low density lipoprotein (mmol/L)	2.6 ± 1.0	3.1 ± 1.1	3.0 ± 0.7
Proportion of hypertension (%)	100.0	100.0	100.0
Proportion of hyperlipidemia (%)	40.0	60.0	46.7
Proportion of hyperglycemia (%)	60.0	40.0	26.7

Data are presented as mean ± standard deviation.

1RM, One-repetition maximum; SBP, systolic blood pressure; DBP, diastolic blood pressure.

### 3.2 Effects of Different Exercise Modes on HR

**FIGURE 1:**
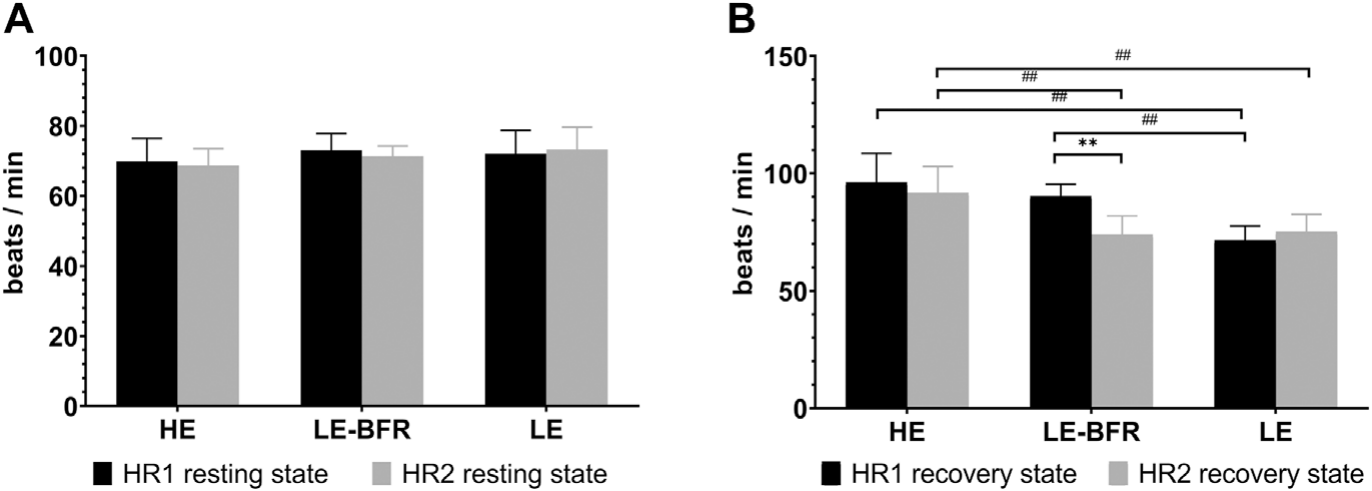
**(A)** HR in the resting state before the first and last training. **(B)** HR in the recovery state after the first and last training. HR1 resting state, HR in the resting state before the first training. HR2 resting state, HR in the resting state before the last training. HR1 recovery state, HR in the recovery state after the first training. HR2 recovery state, HR in the recovery state after the last training. Data are presented as the mean ± standard deviation (SD). **p < 0.01, significantly different from baseline. ##*p* < 0.01, significantly different among groups.

After 12 weeks of training, HR in the resting state didn’t vary significantly in the three groups (*p* > 0.05) (Figure 1A).

After the first training, HR in the recovery state in the LE group (71.5 ± 6.2 b⋅min^−1^) was significantly lower than that in the LE-BFR (90.2 ± 5.2 b⋅min^−1^) and HE groups (96.3 ± 12.3 b⋅min^−1^) (both *p* < 0.01). After 12 weeks of training, HR in the recovery state in the LE-BFR group decreased significantly (*p* < 0.01), and HR in the HE group (91.6 ± 11.4 b⋅min^−1^) was significantly higher than that in the LE-BFR (73.9 ± 8.0 b⋅min^−1^) and LE groups (75.1 ± 7.5 b⋅min^−1^) (both *p* < 0.01) (Figure 1B).

### 3.3 Effects of Different Exercise Modes on BP

**FIGURE 2:**
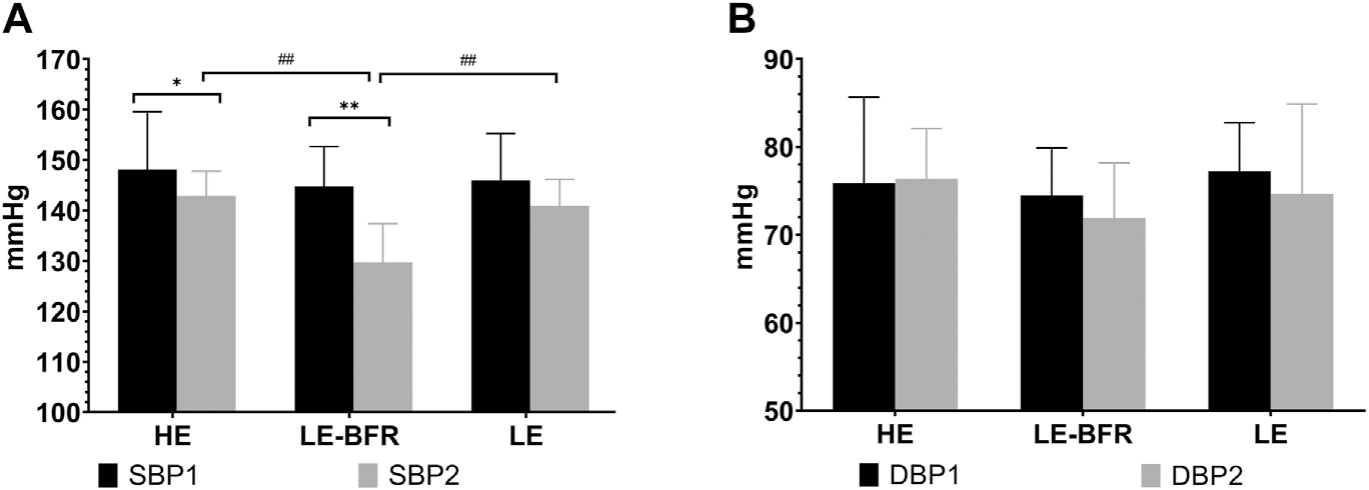
**(A)** SBP in the resting state before the first and the last training. **(B)** DBP in the resting state before the first and the last training. SBP1, SBP before the first training. SBP2, SBP before the last training. DBP1, DBP before the first training. DBP2, DBP before the last training. Data are presented as the mean ± standard deviation (SD). *p < 0.05, **p < 0.01, significantly different from baseline. ##*p* < 0.01, significantly different among groups.

Before the training, there were no significant differences in BP among groups (*p* > 0.05). After 12 weeks of training, SBP in the HE (148.1 ± 11.4 vs. 142.9 ± 4.9 mmHg, *p* < 0.05) and LE-BFR groups (144.7 ± 7.9 vs. 129.7 ± 7.6 mmHg, *p* < 0.01) were significantly decreased compared to that before resistance training. SBP in the LE-BFR group was significantly lower than that in the HE (148.1 ± 11.4 mmHg) and LE groups (140.9 ± 5.2 mmHg) (both *p* < 0.01) (Figure 2A). There were no significant differences in DBP among groups before and after the trainings (*p* > 0.05) (Figure 2B).

### 3.4 Effects of Different Exercise Modes on RMSSD

**FIGURE 3:**
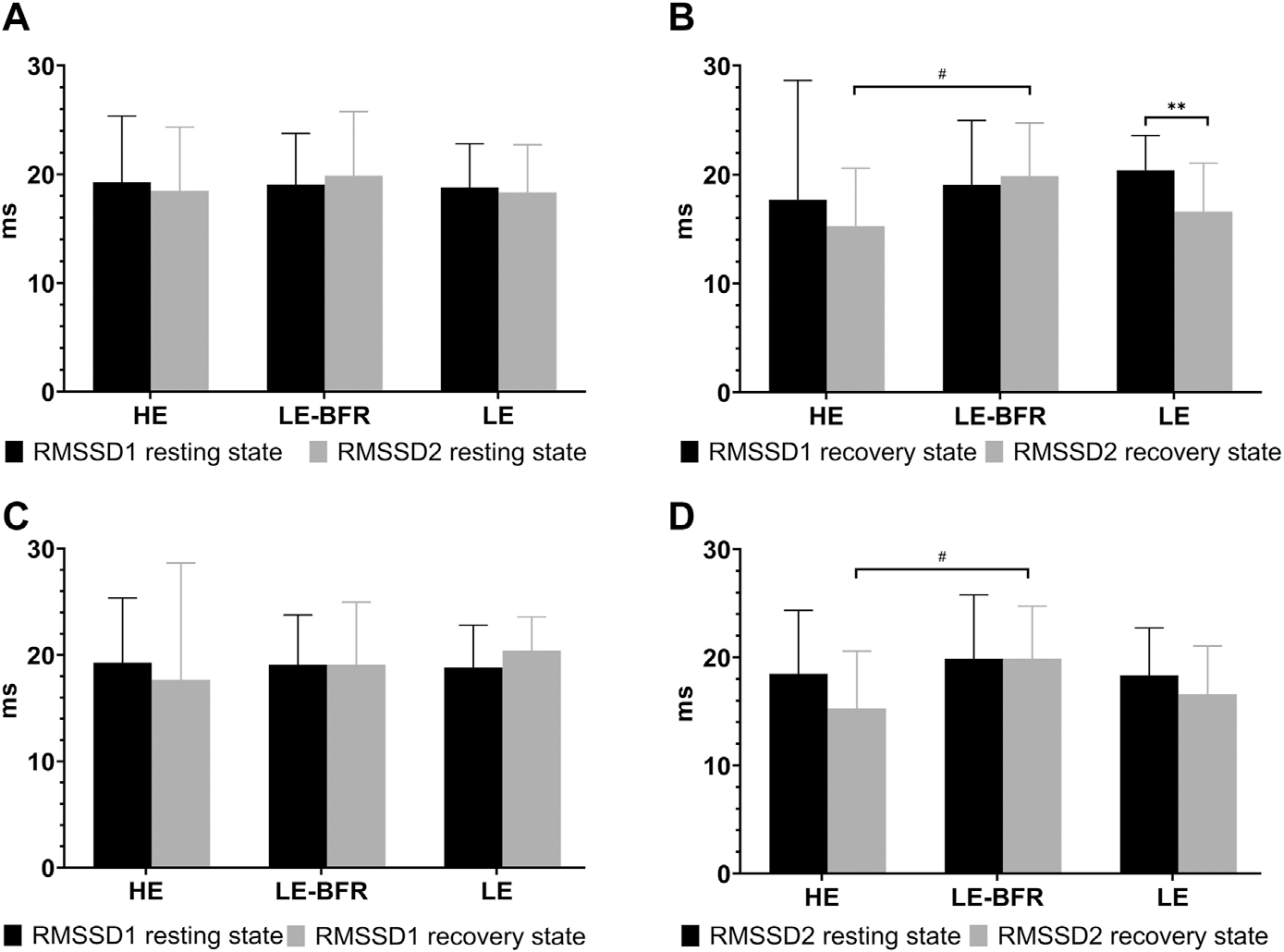
**(A)** RMSSD in the resting state before the first and the last training. **(B)** RMSSD in the recovery state after the first and the last training. **(C)** RMSSD before and after the first training. **(D)** RMSSD before and after the last training. RMSSD1 resting state, RMSSD in the resting state before the first training. RMSSD2 resting state, RMSSD in the resting state before the last training. RMSSD1 recovery state, RMSSD in the recovery state after the first training. RMSSD2 recovery state, RMSSD in the recovery state after the last training. Data are presented as the mean ± standard deviation (SD). **p < 0.01, significantly different from baseline. #*p* < 0.05, significantly different among groups.

In terms of RMSSD, there were no significant differences among groups in the resting states before the training and 12 weeks after the training or in the recovery states after the first training (*p* > 0.05) (Figures 3A, B). After 12 weeks of training, RMSSD in the LE group in the recovery state was decreased significantly (20.4 ± 3.2 vs. 16.7 ± 4.5 m, *p* < 0.01). Comparing the recovery state after the first and last training, there was no significant decrease of RMSSD in the HE group (17.7 ± 11.0 vs. 15.3 ± 5.3 m, *p* > 0.05), and no significant increase of RMSSD in the LE-BFR group (19.1 ± 5.9 vs. 19.9 ± 4.9 m, *p* > 0.05). However, there was significant difference in RMSSD in the recovery state between the HE and LE-BFR groups after the last training (*p* < 0.05) (Figures 3B, D).

### 3.5 Effects of Different Exercise Modes on LF/HF Ratio

**FIGURE 4:**
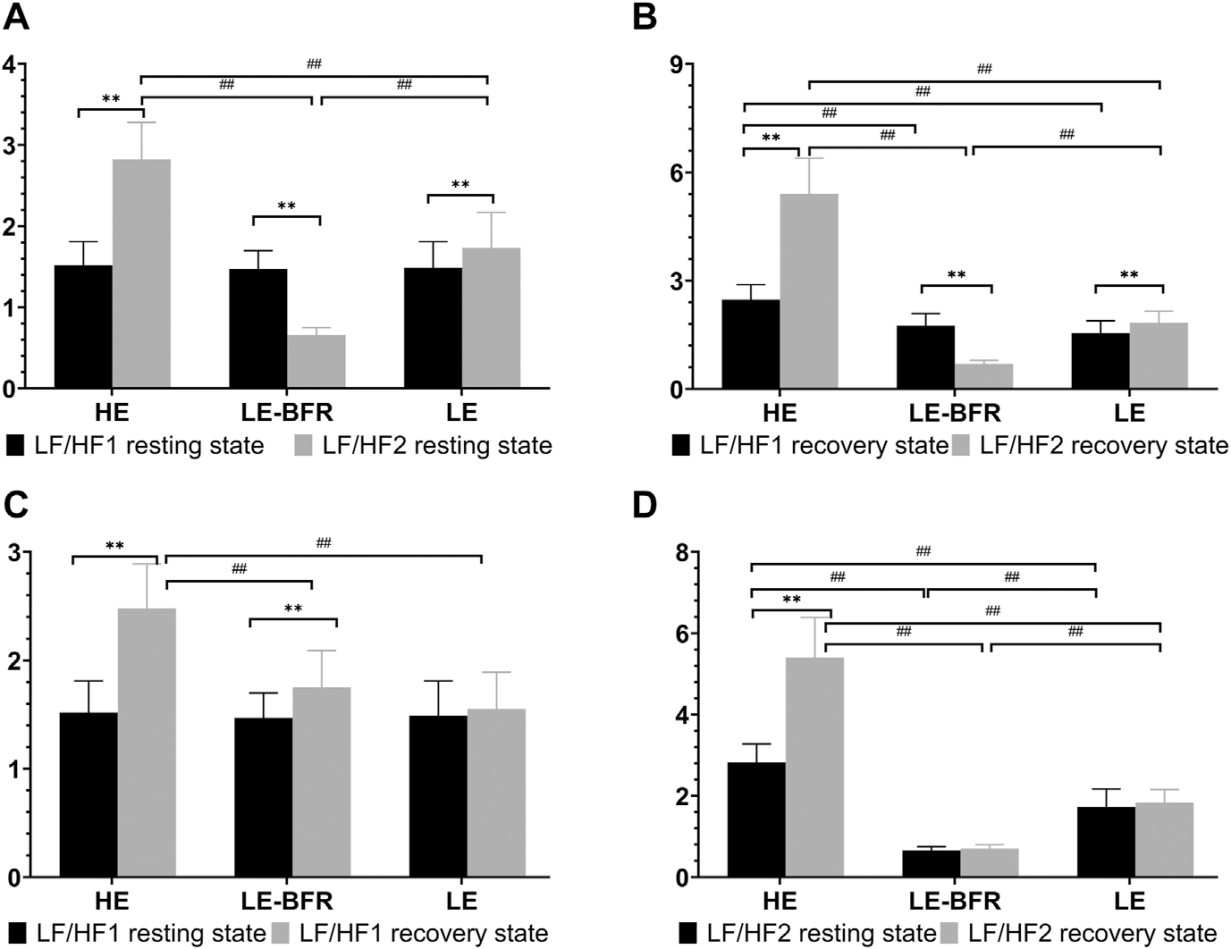
**(A)** LF/HF in the resting state. **(B)** LF/HF in the recovery state. **(C)** LF/HF before and after the first training. **(D)** LF/HF before and after the last training. LF/HF1 resting state, LF/HF in the resting state before the first training. LF/HF2 resting state, LF/HF in the resting state before the last training. LF/HF1 recovery state, LF/HF in the recovery state after the first training. LF/HF2 recovery state, LF/HF in the recovery state after the last training. Data are presented as the mean ± standard deviation (SD). **p < 0.01, significantly different from baseline. ##*p* < 0.01, significantly different among groups.

Before the training, there were no significant differences in LF/HF ratio among groups (*p* > 0.05). After 12 weeks of training, in terms of LF/HF ratio in resting state, significant increases were observed in the HE (1.5 ± 0.3 vs. 2.8 ± 0.5, *p* < 0.01) and LE groups (1.5 ± 0.3 vs. 1.7 ± 0.4, *p* < 0.01), whereas significant decrease was observed in the LE-BFR group (1.5 ± 0.2 vs. 0.7 ± 0.1, *p* < 0.01). Moreover, there were significant differences in LF/HF ratio among groups (*p* < 0.01) (Figure 4A). In the recovery state after the first training, LF/HF ratio in the HE group was significantly higher than that in the LE-BFR and LE groups (both *p* < 0.01) (Figure 4B). In the recovery state after the last training, significant increases in LF/HF ratio were observed in the HE (2.5 ± 0.4 vs. 5.4 ± 1.0, *p* < 0.01) and LE groups (1.6 ± 0.3 vs. 1.8 ± 0.3, *p* < 0.01), while significantly decreased LF/HF ratio was observed in the LE-BFR group (1.8 ± 0.3 vs. 0.7 ± 0.1, *p* < 0.01). There were significant differences in LF/HF ratio among groups (*p* < 0.01) (Figure 4B).

In the recovery state after the first training, compared to the resting state, LF/HF ratios in the HE and LE-BFR groups were increased significantly (both *p* < 0.01). Moreover, LF/HF ratio in the HE group was higher than that in the LE-BFR and LE groups (both *p* < 0.01) (Figure 4C). In the resting state before the last training, LF/HF ratio was increased in the HE group compared to the LE group (*p* < 0.01), and in the LE group compared to the LE-BFR group (*p* < 0.01). In the recovery state after the last training, compared to the resting state before the last training, LF/HF ratio in the HE group was increased significantly (*p* < 0.01), but no significant difference in LF/HF ratio between LE-BFR and LE groups were observed (*p* > 0.05). There were significant differences in LF/HF ratio among the three groups (*p* < 0.01) (Figure 4D). As shown in Table 2, HF in the HE group was decreased very significantly (*p* < 0.01) and HF in the LE-BFR group was increased very significantly (*p* < 0.01) after the training, which led to the highly significant change of LF/HF ratios among the groups.

**TABLE 2:** Parameters of LF and HF in the resting and recovery states.

Variables	Groups	Resting state	Recovery state
LF1 (µn)	HE	577.21 ± 53.92^##^	564 ± 55.61^##^
	LE-BFR	510.56 ± 42.59	498.57 ± 50.82
	LE	502.36 ± 60.51	506.92 ± 47.26
LF2 (µn)	HE	555.96 ± 59.3	567.64 ± 36.08
	LE-BFR	429.29 ± 45.06^##^**	442.02 ± 42.51^##,*^
	LE	559.76 ± 41.96**	566.8 ± 36.07**
HF1 (µn)	HE	386.96 ± 44.68^##^	232.23 ± 36.28^##^
	LE-BFR	352.34 ± 45.31	294.22 ± 57.19
	LE	344.7 ± 38.83	337.85 ± 58.87^##^
HF2 (µn)	HE	199.97 ± 22.7^##^**	107.55 ± 16.18^##^**
	LE-BFR	653.29 ± 50.46^##^**	633.08 ± 57.96^##^**
	LE	335.15 ± 54.92	316.82 ± 49.32

LF1, LF, of the first training; LF2, LF, of the last training; HF1, HF, of the first training; HF2, HF, of the last training.

Data are presented as the mean ± standard deviation.

*p < 0.05 vs. baseline, **p < 0.01 vs. baseline.

^##^ *p*<0.01 among groups.

## 4 Discussion

This study aimed to find out the resistance exercise type that was beneficial for regulating BP and ANS, and that could avoid higher cardiovascular risk. It was suggested that compared to HE and LE, LE-BFR could more effectively regulate SBP and the imbalance and dysregulation of the ANS in hypertension patients. It was also indicated that when hypertension patients used resistant exercise to decrease SBP in the resting state, LE-BFR was the most effective exercise type compared to HE and LE, leading to less cardiovascular risk.

Both high BP and high HR are risk factors of cardiovascular, however, this research didn’t find the decrease of SBP related to HR in the resting states. In this research, there was no significant difference between the resting HR before and after the training.

HR recovery rate reflects the balance of the autonomic nervous system (Silva et al., 2021). Previous studies have found slow HR recovery rate after exercise is associated with increased risk for arrhythmia and other cardiovascular morbidities and mortality, while increased HR recovery rate is associated with improved prognosis and lower mortality related to cardiovascular disease (Heffernan et al., 2007; Smith et al., 2005; Stein et al., 2005; Task Force, 1996; Tsuji et al., 1996; Zhang et al., 2016). Cardiovascular and neurovegetative adaptations to exercise training through alteration in sympathovagal balance lead to changes in HR recovery (Suzic et al., 2017). After the first training, the HR recovery rate of the HE and LE-BFR groups was slower than that of the LE group, implying that for hypertension patients without exercise habits, the cardiovascular risk caused by temporary HE and LE-BFR was higher than that by LE. After 12 weeks of training, the HR recovery rate of the LE-BFR group was significantly improved, and there was no difference in this value between the LE and LE-BFR group. In addition, the HR of HE group was even higher than that of the LE-BFR and LE groups. This finding indicated that LE-BFR could result in better adaptability of the cardiovascular function, thereby lowering cardiovascular risk. However, HE could cause higher cardiovascular risks compared to LE-BFR and LE.

High SBP is the leading cause of death and disability worldwide (Lim et al., 2012). The meta-analysis by Naci et al. (2019) pointed out that middle to high intensity resistance exercises could effectively reduce BP. Consistent with their research, the present study suggested that HE could effectively decrease SBP. Furthermore, our research found LE-BFR could also decrease SBP, and the effect of LE-BFR was more significant compared to the HE and LE modes. These results demonstrated that LE-BFR could exert better antihypertensive effects in subjects compared to HE. For people who are not able to perform high intensity resistant exercises, they can reduce their BP by LE-BFR training.

RMSSD is a parameter to evaluate parasympathetic activity. In the present study, RMSSD in the resting state didn’t change significantly. This result was similar to the findings from several previous studies. The study by Takahashi et al. (2009) included 17 healthy old men who conducted high intensity resistant exercises for 12 weeks and showed that RMSSD in the resistant exercise group didn’t change significantly. Millar et al. (2013) suggested similar results of RMSSD in the resting state with our findings when they inspected elderly hypertension patients who performed low intensity resistant exercises for 12 weeks. The findings about RMSSD by Gerage et al. (2013) also consistent with our results. The difference between us is their subjects were elderly healthy women but our subjects were elderly hypertension patients. However, the conclusions of the study by Caruso et al. (2015) on RMSSD in the resting state in elderly patients with coronary heart disease was different with our results. They showed that RMSSD in resting state had increased after low intensity resistant exercises for 8 weeks. The cause of the difference might be the different exercise forms, exercise volume and detection time.

In the present study, the RMSSD in the recovery state after the first training was the highest in the LE group, and the lowest in the HE group. Although there were no significant differences among groups, the trend was consistent with the result of the transient study by Okuno et al. (2014), which covered about 20–29 years healthy men. In their study, there were significant differences among groups. The possible reason of significant differences in their study was that the exercise intensity of their subjects was 10% higher than ours, and the subjects must exercise to exhaustion in the last set. The article by Isidoro et al. (2017) on healthy men aged 20–35 years also showed that transient high intensity resistant exercises could reduce RMSSD significantly. The difference between their and our results might come from the fact that all limbs of subjects in their research should exercise and the total exercise volume was different. Taken together, it was suggested that RMSSD could not be used as an independent indicator to evaluate cardiovascular risk. RMSSD might indicate an increase in cardiovascular risk only when other parameters of the ANS prompted this risk with decreasing RMSSD.

LF is a parameter to express sympathetic and vagal modulations simultaneously, and this index represents sympathetic modulations best. HF is an indicator of vagal modulation. LF/HF ratio is a parameter to evaluate the sympathovagal balance (Task Force, 1996). Enhanced sympathetic and weakened parasympathetic predominance regulations are associated with increased cardiovascular risk (Mourot et al., 2004; Seiler et al., 2007; Lima et al., 2011). In the present study, after 12 weeks of training, both in the resting state and the recovery state, LF/HF ratio in the HE and LE groups increased significantly, whereas this value in the LE-BFR group decreased significantly. This indicated that patients in the LE-BFR group had significantly improved ANS, while those in the HE and LE groups didn’t show this advantage. The study of Lima et al. (2011) about HE only also showed that LF/HF ratio increased after HE. However, the study by Bellavere et al. (2018) showed that after HE for 4 months, the LF/HF ratio of subjects with type II diabetes decreased. The transient high-intensity resistance training proposed by Isidoro et al. (2017) also showed a decreased LF/HF ratio. The difference might come from different modes of exercise, which was supported by the study by Morishima et al. (2018). They compared low-intensity high-repetition and high-intensity low-repetition resistance exercises, and the results verified that the intensity, number of repetition, and intermittent resting time were important factors affecting BP and endothelial function after exercise. However, their study didn’t involve the indicators for ANS. Moreover, the difference in detection time might also affect the results.

Although LF/HF ratios in both HE and LE groups increased significantly, there was also difference between them. LF increased significantly in the LE group while HF decreased significantly in the HE group after 12 weeks of training. The LF/HF ratio in HE group increased greatly (1.52–2.82 in the resting state; 2.48 to 5.40 in the recovery state), but the LF/HF ratio in LE group was still in normal range (1.49–1.73 in the resting state; 1.55 to 1.83 in the recovery state). Because the long-term follow-up study about heart rate variability and mortality (Kuo et al., 2018) demonstrated high LF/HF ratio was an independent risk factor for mortality and LF/HF ratio >2.6 was associated with a higher mortality, our research indicates LE and LE-BFR was safer than HE.

This study was the first to compare BP and ANS in hypertension patients after performing three types of resistant exercises for 12 weeks. Those are different from others. We found LE-BFR had the most obvious effect on reducing SBP and adjusting sympathetic-parasympathetic balance, and was associated with lower cardiovascular risk compared to HE and LE. There are some limitations in the present study. For example, BP in the recovery state wasn’t measured. Additionally, although the effects of 12 weeks of training was examined, the long-term effects of resistance exercise needed to be further observed. Furthermore, no biochemical indicators related to ANS were detected, making it difficult to identify the causes of the changes in RMSSD, LF and HF.

## Data Availability

The raw data supporting the conclusions of this article will be made available by the authors, without undue reservation.
